# Plastome Evolution and Codon Usage Dynamics in *Argentina* (Rosaceae): Phylogenomic Resolution and Adaptive Signatures

**DOI:** 10.1002/ece3.74073

**Published:** 2026-07-27

**Authors:** Guang‐Nan Zhang, Li‐Zhen Ling, Jie Tian, Shu‐Dong Zhang

**Affiliations:** ^1^ Key Laboratory of Qinghai‐Tibetan Plateau Biotechnology of Ministry of Education Academy of Agriculture and Forestry Sciences of Qinghai University (Qinghai Academy of Agriculture and Forestry Sciences) Xining China; ^2^ Key Laboratory for Specialty Agricultural Germplasm Resources Development and Utilization of Guizhou Province Liupanshui Normal University Liupanshui China

## Abstract

The genus *Argentina* (Rosaceae) exhibits extensive morphological plasticity that has long complicated taxonomic delimitation and phylogenetic inference. Here, we report complete chloroplast genome sequences for three species— *A. stenophylla* var. *stenophylla*, *A. anserina* , and *A. leuconota* var. *leuconota*—and integrate them with 15 previously published congeneric plastomes to investigate genome architecture, phylogenetic relationships, and synonymous codon usage bias (CUB). All 18 plastomes retain the canonical quadripartite structure and near‐identical gene complements, underscoring extraordinary architectural stability. Phylogenomic reconstruction strongly supports the monophyly of *Argentina* and resolves five well‐supported clades, corroborating the transfer of *Sibbaldia micropetala* and *S. phanerophlebia* into *Argentina*. Codon analysis revealed that the effective number of codons (ENC) is consistently high (~48), while the codon adaptation index (CAI) remains uniformly low (~0.17), together indicating weak overall codon bias. Neutrality, ENC, and PR2 analyses converge to indicate that natural selection—not mutational pressure—is the principal force shaping CUB, accounting for over 95% of observed variation. Although hierarchical clustering of RSCU values largely recapitulates the plastome phylogeny, discrete departures in specific lineages suggest that ecological selection may have independently modulated codon optima. Notably, only two amino acids (Gln and Gly) retain universally conserved optimal codons, whereas seven amino acids exhibit dual optimal codons with pronounced interspecific variation. These findings reveal a decoupled evolutionary architecture: while genome organization is ultraconserved, synonymous sites undergo ongoing selective refinement. This dissociation between rigid genomic architecture and malleable codon configurations might point to a coevolutionary process in which plastid translational machinery and environmental selection reciprocally fine‐tune synonymous codon choice, shedding light on how *Argentina* modulates organellar gene expression to cope with environmental heterogeneity across the Sino‐Himalayan region and the Malay Archipelago.

## Graphical abstract

This study assembled chloroplast genomes for three *Argentina* species and compared them with 15 others to resolve long‐standing taxonomic issues. Phylogenomic analyses strongly supported the monophyly of *Argentina* and identified five major clades, while analyses of codon usage revealed that natural selection is the predominant force shaping codon evolution. Notably, optimal codon repertoires exhibited both deeply conserved patterns and species‐specific variations, and clustering based on codon usage was discordant with the chloroplast phylogeny, suggesting lineage‐specific adaptive fine‐tuning.

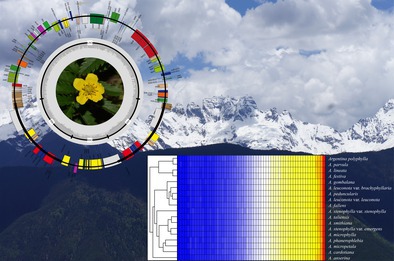

## 1 Introduction

The genus *Argentina* Hill (Rosaceae: Rosoideae, Potentilleae) comprises approximately 75 species primarily distributed across the Sino‐Himalayan region and the Malay Archipelago, exhibiting high economic value and remarkable ecological adaptability (Soják 2010; Li, Khasbagan, et al. 2024). This broad ecological amplitude and geographical range have fostered pronounced morphological plasticity and convergent evolution, frequently rendering *Argentina* species superficially similar to congeners in *Potentilla* and *Sibbaldia* and thereby generating persistent taxonomic ambiguity (Ikeda and Hideaki 1993). Although Hill (1756) first segregated *Argentina* from *Potentilla* (Hill et al. 1756), and Rydberg (1898, 1908) subsequently upheld its generic status based on style position (lateral in *Argentina* versus subterminal in *Potentilla s.s*.) (Rydberg 1898, 1908), this circumscription was not widely accepted because some Asian species of *Argentina* exhibit intermediate morphological characters (Soják 2010). It was not until Soják (2010) identified a stable difference in stipular auricle position—ventral in *Argentina* versus lateral in *Potentilla s.s*.—that a robust morphological basis for the generic independence of *Argentina* was established (Soják 2010).

However, morphological characters alone are insufficient to resolve interspecific relationships within *Argentina* or to clarify its phylogenetic affinities with allied genera, rendering molecular evidence indispensable (Feng et al. 2017; Dobe and Paule 2010). Early phylogenetic studies employing chloroplast DNA fragments (e.g., *matK*, *trnL‐F*) and nuclear ribosomal ITS sequences began to elucidate the evolutionary framework of the tribe Potentilleae; however, conflicting topologies regarding the monophyly of *Argentina* and its relationships with related genera persisted (Dobe and Paule 2010; Eriksson et al. 2015). With the advent of high‐throughput sequencing, complete chloroplast genomes have furnished abundant phylogenetic signals capable of resolving these controversies. Phylogenomic analyses have confirmed the division of Potentilleae into two subtribes, Fragariinae and Potentillinae, with *Argentina* forming a stable sister group to *Potentilla s.s*. within Potentillinae (Li, Khasbagan, et al. 2024; Li, Zhang, et al. 2024). Molecular data further indicate that *Sibbaldia micropetala* and *S. phanerophlebia* should be transferred to *Argentina* as *A. micropetala* and *A. phanerophlebia*, respectively (Feng et al. 2017; Eriksson et al. 2015); moreover, representatives of *Tylosperma* and *Piletophyllum* are nested within *Argentina*, corroborated by their shared possession of ventral stipular auricles (Soják 2010). Within the genus, chloroplast genome analyses have revealed a fully resolved phylogeny comprising four distinct clades: the earliest‐diverging *A. anserina* – *A. smithiana* lineage, the sister pair *A. micropetala*–*A. phanerophlebia*, the *A. stenophylla* – *A. microphylla* –*A. taliensis*–*A. tatsienluensis* clade, and the *A. fallens*–*A. peduncularis*–*A. leuconota*–*A. cardotiana* clade (Li, Khasbagan, et al. 2024; Li, Zhang, et al. 2024).

Beyond their phylogenetic utility, chloroplast genomes provide an exceptional model for investigating synonymous codon usage bias (CUB), a central issue in evolutionary genomics that offers critical insights into the neutralist–selectionist debate. Although neutral theory attributes most molecular variation to genetic drift (Kimura 1983), the discovery of optimized codon usage in highly expressed genes across diverse organisms provided early empirical evidence for weak translational selection (Toshimichi 1981; Sharp and Wen‐Hsiung 1987), thereby stimulating integrative theoretical frameworks such as the selection–mutation–drift model (Bulmer 1991). Synonymous codons are functionally consequential: optimal codons correspond to abundant tRNA pools, thereby enhancing translational efficiency and accuracy (Toshimichi 1981; Sharp and Wen‐Hsiung 1987), while codon choice also influences mRNA stability (Presnyak et al. 2015) and co‐translational protein folding kinetics (Plotkin and Kudla 2011), reflecting adaptive optimization to cellular environments and effective population sizes. Furthermore, CUB patterns can serve as informative markers in phylogenetic and comparative genomic analyses because closely related species often retain similar usage patterns due to shared ancestry and common selective regimes (Plotkin and Kudla 2011). Chloroplast genomes are particularly valuable models for CUB analysis due to their conserved architecture. They consistently exhibit codon biases ending in A/U. These biases are shaped by the interplay of mutational pressure and natural selection (Morton 1993; Ravi et al. 2008). In chloroplast genomes, natural selection frequently predominates over mutation. This predominance provides a framework for detecting adaptive evolution in photosynthesis‐related genes. Nevertheless, patterns of CUB within *Argentina* and their potential correlation with phylogenetic relationships remain entirely unexplored.

To address this gap, we sequenced and assembled the complete chloroplast genomes of three representative species— *A. stenophylla* var. *stenophylla*, *A. anserina* , and *A. leuconota* var. *leuconota*—and performed a comprehensive comparative analysis of chloroplast genome features across 18 species of *Argentina*. Phylogenomic reconstruction based on 21 taxa (18 *Argentina* species and three *Potentilla* outgroups) provided a robust framework for inferring evolutionary relationships. Within this phylogenetic context, we conducted a multifaceted CUB analysis to assess whether codon usage patterns correlate with the evolutionary trajectories of these species. By integrating comparative genomics and phylogenomics, this study provides novel insights into the mechanisms shaping organellar genome function, species‐level adaptive evolution, and taxonomic revision within *Argentina*.

## 2 Materials and Methods

### 2.1 Plant Materials, DNA Extraction, Sequencing, and Assembly

Fresh leaves from three *Argentina* species ( *A. anserina* , *A. leuconota* var. *leuconota*, and *A. stenophylla* var. *stenophylla*) were collected from various provinces in China (Table S1) and rapidly dried using silica gel. Species identification was confirmed by Prof. Shu‐Dong Zhang, and voucher specimens were deposited at the Herbarium of Kunming Institute of Botany (KUN). Total genomic DNA was extracted using a modified CTAB method. DNA libraries were constructed and subjected to paired‐end sequencing (PE150) on the Illumina NovaSeq platform. High‐quality reads were utilized for *de novo* assembly of chloroplast genomes using SPAdes v3.15 (Prjibelski et al. 2020). The resulting contigs were visualized and circularized using Bandage (Wick et al. 2015). Complete chloroplast genomes were annotated using the Plastid Genome Annotator (PGA) (Qu et al. 2019), referencing genomes of related *Argentina* species. Genome maps were generated using OGDRAW (Stephan et al. 2019).

### 2.2 Phylogenetic Analysis of *Argentina*

A total of 21 chloroplast (cp) genomes, including 18 species and varieties from *Argentina* and three *Potentilla* species as outgroups (Table S1), were incorporated into the phylogenetic analysis. Whole chloroplast genome sequences were aligned using MAFFT v6.833 (Katoh et al. 2005). Phylogenetic trees were reconstructed employing both Bayesian Inference (BI) and Maximum Likelihood (ML) methods, adhering to previously established protocols (Zhang, Yan, and Ling 2023). For ML analysis, we used RAxML v7.2.64 under the GTRGAMMA model with 1000 bootstrap replicates. BI was conducted using MrBayes v3.1.2. The Markov Chain Monte Carlo (MCMC) analysis ran for 100,000 generations, with trees sampled every 100 generations. The first 25% of trees were discarded as burn‐in, and a majority‐rule consensus tree was generated from the remaining trees, with posterior probability (PP) values calculated for each node.

### 2.3 Codon Usage Analysis

Coding sequences (CDS) were extracted from the chloroplast genomes of 18 *Argentina* samples, with duplicated genes and pseudogenes excluded. Only CDS longer than 300 bp and containing standard start (ATG) and stop (TAA, TAG, or TGA) codons were retained for further analysis. Nucleotide composition indices, including GC content at the first, second, and third codon positions (GC1, GC2, GC3) as well as overall GC content (GCall), were computed using EMBOSS‐cusp (https://www.bioinformatics.nl/cgi‐bin/emboss/cusp). Additionally, the Codon Adaptation Index (CAI), Relative Synonymous Codon Usage (RSCU), and the Effective Number of Codons (ENC) were calculated using CodonW version 1.4.2 (Fuglsang 2006; Wright 1990). Codons with RSCU > 1 were considered preferentially used, while an ENC value of 35 or less indicated significant codon usage bias (Liu et al. 2010; Duret 2000; Niu et al. 2021).

### 2.4 Sources of Codon Usage Bias

Neutrality plots (GC12 vs. GC3) were constructed to evaluate the influence of mutation pressure relative to natural selection (Kawabe and Miyashita 2003). In this framework, the first and second codon positions (GC12) are strongly constrained by functional selection because nonsynonymous mutations at these sites often alter amino‐acid identity and protein function, whereas the third position (GC3) is largely free to vary and primarily records the background mutational bias. Consequently, a regression slope approaching 1 indicates that GC12 and GC3 change in concert, implying that genome‐wide mutation pressure is the principal driver of nucleotide composition; conversely, a slope near 0 reflects a decoupling of GC3 from the functionally constrained GC12, signifying that natural selection dominates codon usage by restricting variation at the first two positions while allowing GC3 to respond to other evolutionary forces (Li et al. 2022).

ENC plots were generated by comparing the observed ENC values against the expected values under neutral evolution, calculated using the formula ENC_exp = 2 + GC3 + 29/[GC3^2^ + (1 − GC3)^2^] (Parvathy et al. 2022). Under a purely mutational model, codon bias should be fully explained by genomic GC content. Therefore, observed ENC values are expected to fall on or near this curve. Systematic deviations below the curve indicate that the observed bias is stronger than mutation alone can account for. Such deviations reveal additional selective constraints acting on synonymous codon choice. The most common constraint is translational selection. (Fuglsang 2008).

Additionally, Parity Rule 2 (PR2) analysis (Sueoka 1999) was applied to examine strand‐specific compositional bias, by testing whether A equals T and G equals C at the third codon position. In the absence of directional selection, mutational equilibrium should satisfy this parity rule, causing points to cluster around the centroid (0.5, 0.5); asymmetric dispersion along the A/(A + T) or G/(G + C) axes therefore signals departures from neutral mutation and implicates lineage‐specific or strand‐specific selective pressures.

### 2.5 Optimal Codon Identification and RSCU Clustering

In this study, we employed ENC as a proxy for expression level, based on the well‐established inverse relationship between ENC and codon bias strength: highly expressed genes typically exhibit stronger translational selection and thus lower ENC values (more biased usage), whereas lowly expressed genes display higher ENC values. Accordingly, genes were ranked by their ENC values, and those falling in the bottom 10% (lowest ENC) and top 10% (highest ENC) were designated as high‐expression and low‐expression proxies, respectively. The difference in RSCU, defined as ΔRSCU = RSCU_high − RSCU_low, was calculated for each codon. Optimal codons were identified as those satisfying two criteria: an RSCU value > 1.0 in the high‐expression proxy set and a ΔRSCU value of at least 0.08 relative to the low‐expression proxy set (Xiang et al. 2015). Based on RSCU values of 18 *Argentina* samples, hierarchical cluster analysis was performed via average linkage clustering with Pearson correlation distance in OriginPro 2025b (OriginLab Corporation, Northampton, MA, USA).

## 3 Results

### 3.1 Structural Conservation and Features of *Argentina* Chloroplast Genomes

**FIGURE 1:**
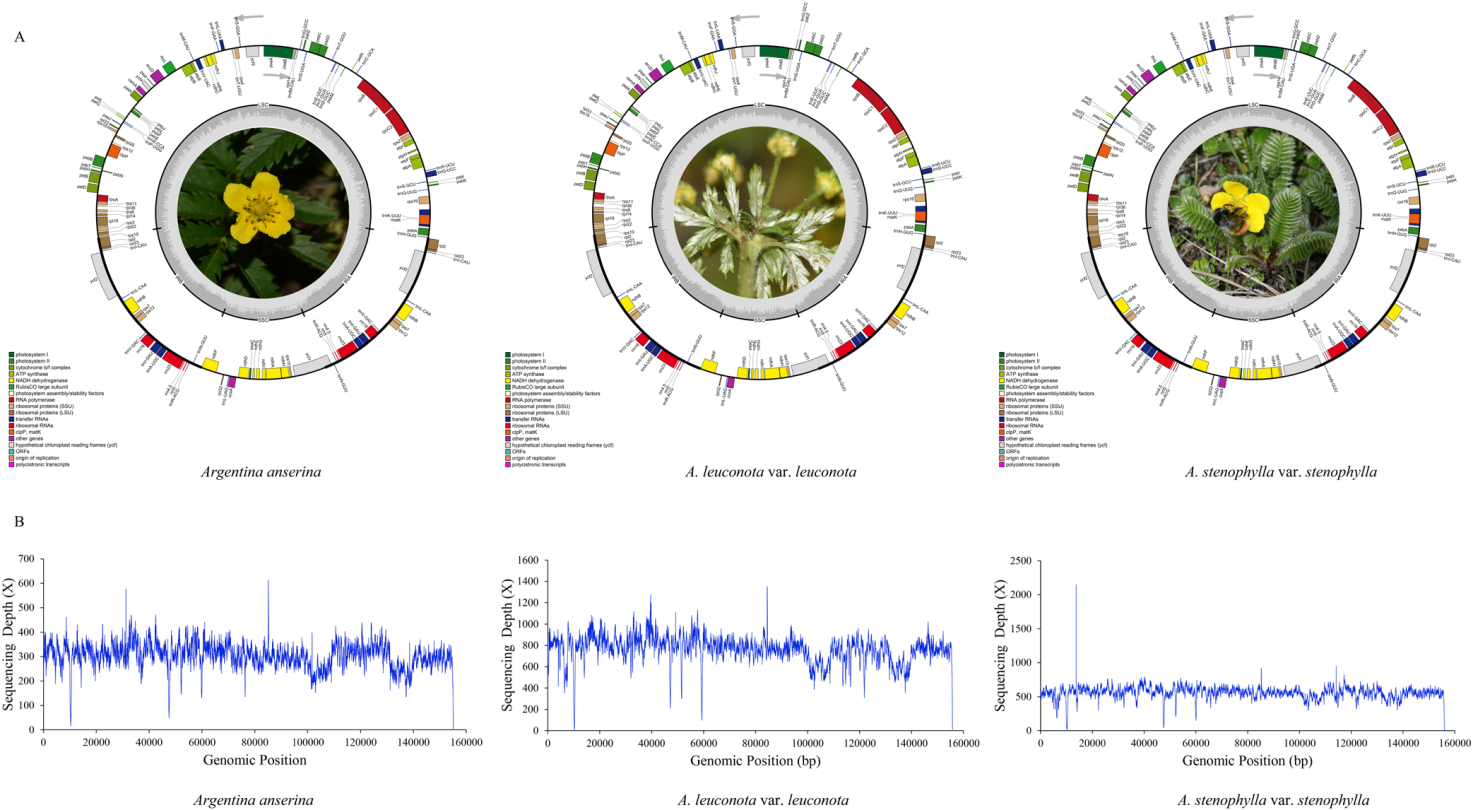
Chloroplast genome map of three *Argentina* species (A) and their sequencing depths (B). The gray inside circle indicates the GC level of every genomic position. Genes inside in the outer circle of genomic map are transcribed clockwise and vice versa. The different functional gene categories are shown in the different colors.

The newly assembled chloroplast genomes of three *Argentina* species: *A. anserina* , *A. leuconota* var. *leuconota*, and *A. stenophylla* var. *stenophylla* exhibit the highly conserved structural features typical of most angiosperms (Figure 1A). These genomes range from 155,148 bp in *A. anserina* to 155,972 bp in *A. stenophylla* var. *stenophylla*, with an average sequencing depth of 303.86×, 764.21× and 557.59× for *A. anserina* , *A. leuconota* var. *leuconota*, and *A. stenophylla* var. *stenophylla*, respectively (Table 1 and Figure 1B). All three cp genomes display the canonical quadripartite structure, consisting of a large single‐copy (LSC) region, a small single‐copy (SSC) region, and a pair of inverted repeats (IRs) (Figure 1A). The sizes of these regions are comparable to those of other *Argentina* species, with LSC regions spanning 84,425–86,350 bp, SSC regions 17,769–18,911 bp, and IRs 25,702–26,725 bp (Table 1). In addition, the overall GC content among 18 *Argentina* genomes remains low and stable, varying from 36.62% to 37.15% (Table 1), consistent with the AT‐rich bias characteristic of plant chloroplast genomes.

**TABLE 1:** Information of complete plastomes of *Argentina* species.

Species	Accession number	Gene number			Length (bp)				GC content (%)			
		PCG	rRNA	tRNA	Genome	LSC	SSC	IR	Genome	LSC	SSC	IR
***Argentina anserina***	PX057649	84	8	37	155,148	85,068	18,676	25,702	36.74	34.52	30.71	42.60
*A. cardotiana*	OR863689	84	8	37	156,725	86,049	18,704	25,986	37.01	34.85	31.10	42.72
*A. fallens*	OR863690	84	8	37	155,513	85,218	17,861	26,217	36.94	34.86	30.89	42.37
*A. festiva*	OR863691	84	8	37	156,980	86,165	18,837	25,989	36.67	34.44	30.46	42.63
*A. gombalana*	MW307907	84	8	37	156,059	85,618	18,489	25,976	37.02	34.85	31.03	42.72
***A. leuconota* var. *leuconota***	PX057650	84	8	37	155,709	84,425	17,892	26,696	37.06	35.02	31.03	42.31
*A. leuconota* var. *brachyphyllaria*	OR863693	84	8	37	155,650	84,431	17,769	26,725	37.04	34.97	31.09	42.28
*A. lineata*	OQ992657	84	8	37	157,093	86,350	18,773	25,985	36.62	34.35	30.44	42.64
*A. micropetala*	OR863696	84	8	37	156,563	85,947	18,608	26,004	37.07	34.90	31.27	42.72
*A. microphylla*	OR863698	84	8	37	156,563	85,855	18,782	25,963	37.10	34.98	31.17	42.76
*A. parvula*	OR863707	84	8	37	156,318	85,493	18,911	25,957	37.04	34.93	30.91	42.74
*A. peduncularis*	OR863699	84	8	37	155,650	84,442	17,794	26,707	37.05	34.98	31.04	42.31
*A. phanerophlebia*	OR863700	84	8	37	155,504	85,619	18,461	25,712	37.11	34.98	31.16	42.79
*A. polyphylla*	OR863702	84	8	37	156,718	86,061	18,731	25,963	37.00	34.81	31.08	42.76
*A. smithiana*	MW307911	84	8	37	156,753	86,034	18,719	26,000	37.03	34.88	31.10	42.73
** *A. stenophylla* var. *stenophylla***	PX057651	84	8	37	155,972	85,222	18,766	25,992	37.15	35.02	31.18	42.79
*A. stenophylla* var. *emergens*	OR863706	84	8	37	156,572	86,049	18,589	25,967	37.06	34.88	31.21	42.77
*A. taliensis*	OR863704	84	8	37	156,522	85,872	18,656	25,997	37.09	34.94	31.16	42.75

*Note:* The species with bold indicates the new sequenced one in this study.

Annotation revealed an identical complement of 129 genes in each plastome: 84 PCGs, 37 tRNA genes, and 8 rRNA genes (Table 2). These genes are organized into functional categories responsible for self‐replication (e.g., ribosomal proteins, RNA polymerase), photosynthesis (e.g., *psa*, *psb*, *pet*, *atp*, *rbcL* genes), other functions (e.g., *matK*, *accD*), and a few of unknown function. The order and arrangement of these genes are perfectly collinear across the three species (Figure 1A), underscoring a profound structural stasis in the chloroplast genome of *Argentina*. This extreme conservation limits the utility of structural rearrangements as phylogenetic markers but highlights the strong evolutionary constraints maintaining the core photosynthetic machinery.

**TABLE 2:** The gene information of *Argentina* species chloroplast genomes.

Function of genes	Group of genes	Gene name
Photosynthesis	Photosystem I	*psaA*, *psaB*, *psaC*, *psaI*, *psaJ*
	Photosystem II	*psbA*, *psbB*, *psbC*, *psbD*, *psbE*, *psbF*, *psbH*, *psbI*, *psbJ*, *psbK*, *psbL*, *psbM*, *psbN*, *psbT*, *psbZ*
	Cytochrome b/f complex	*petA, petB* ^a^, *petD* ^a^, *petG, petL, petN*
	ATP synthase	*atpA, atpB, atpE, atpF, atpH, atpI*
	NADH‐dehydrogenase	*ndhA* ^a^, ndhB*^a^, *ndhC, ndhD, ndhE, ndhF, ndhG, ndhH, ndhI, ndhJ, ndhK*
	Large subunit Rubisco	*rbcL*
Protein synthesis and DNA‐replication	Subunits of RNA polymerase	*rpoA, rpoB, rpoC1* ^a^, *rpoC2*
	Ribosomal protein small subunit	*rps2*, *rps3*, *rps4*, rps7*, *rps8*, *rps11*, rps12*^b^, *rps14*, *rps15*, *rps16* ^a^, *rps18*, *rps19*
	Ribosomal protein large subunit	rpl2*^a^, *rpl14*, *rpl16* ^a^, *rpl20*, *rpl22*, rpl23*, *rpl32*, *rpl33*, *rpl36*
	Transfer RNAs	trnA‐UGC*^a^, *trnC‐GCA*, *trnD‐GUC*, *trnE‐UUC*, *trnF‐GAA*, *trnfM‐CAU*, *trnG‐GCC*, *trnG‐UCC* ^a^, *trnH‐GUG*, trnI‐CAU*, trnI‐GAU*^a^, *trnK‐UUU* ^a^, trnL‐CAA*, *trnL‐UAA* ^a^, *trnL‐UAG*, *trnM‐CAU*, trnN‐GUU*, *trnP‐UGG*, *trnQ‐UUG*, trnR‐ACG*, *trnR‐UCU*, *trnS‐GCU*, *trnS‐GGA*, *trnS‐UGA*, *trnT‐GGU*, *trnT‐UGU*, trnV‐GAC*, *trnV‐UAC* ^a^, *trnW‐CCA*, *trnY‐GUA*
	Ribosomal RNAs	rrn4.5*, rrn5*, rrn16*, rrn23*
Other genes	Maturase	*matK*
	C‐type cytochrome synthesis gene	*ccsA*
	Subunit of Acetyl‐CoA‐carboxylase	*accD*
	Envelope membrane protein	*cemA*
	ATP‐dependent protease	*clpP* ^a^
Genes of unknown function	Conserved hypothetical gene	*ycf1*, ycf2*, *ycf3* ^b^, *ycf4*

*Note:* * indicates the number of repeat units is 2.

^a^ Gene contains a single intron.

^b^ Gene contains two introns.

### 3.2 Phylogenomic Analysis of *Argentina* Species

**FIGURE 2:**
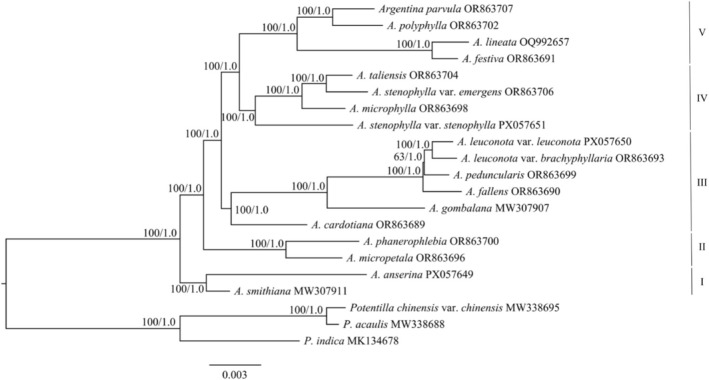
The phylogenetic tree of eighteen *Argentina* species based on cp genome sequences. Numbers at nodes correspond to maximum likelihood bootstrap percentage and the posterior probability of Bayesian inference.

We reconstructed the phylogeny of the genus *Argentina* using complete chloroplast genome sequences from 18 samples, with three *Potentilla* species serving as outgroups (Figure 2). The results strongly supported the monophyly of the genus (PP = 1, BS = 100). Within the genus, five distinct clades were identified. *Argentina anserina* formed a basal sister taxon to *A. smithiana* (Clade I, PP = 1, BS = 100), with these two species being most closely related to *A. micropetala* and *A. phanerophlebia* constituted a separate branch (Clade II). *Argentina leuconota* var. *leuconota* and five other *Argentina* species formed one well‐supported branch (Clade III). The remaining species were divided into two major clades: *A. stenophylla* var. *stenophylla* clustered closely with *A. microphylla* , *A. stenophylla* var. *emergens*, and *A. taliensis* in another branch (Clade IV). The other four species: *A. festiva* , *A. lineata* , *A. polyphylla* , and *A. parvula* formed the sister clade (Clade V). The robust placement of *A. micropetala* and *A. phanerophlebia* within *Argentina* unequivocally supports their transfer from *Sibbaldia*, corroborating earlier proposals based on stipular auricle morphology and fragment‐based molecular data (Feng et al. 2017; Eriksson et al. 2015). By contrast, our whole‐plastome analysis resolves relationships that were previously ambiguous or conflicting in studies employing individual plastid markers (e.g., *matK*, *trnL‐F*) or nuclear ITS sequences (Dobe and Paule 2010; Eriksson et al. 2015): whereas earlier fragment‐based topologies often yielded weak support for the placement of these species, our data provide strong resolution.

### 3.3 Analysis of Codon Usage Patterns Among *Argentina* Species

The 53 conserved protein‐coding genes across eighteen *Argentina* samples were filtered for codon usage analysis. The results reveal distinct and shared patterns in synonymous codon usage among these species (Table 3). The overall GC content of these coding sequences (GCall) is uniformly low, ranging narrowly from 38.30% to 38.58%. A notable pattern emerges at the three codon positions: GC1 (46.89%–47.14%) > GC2 (39.28%–39.47%) > GC3 (28.63%–29.17%). This gradient, characterized by the third position being the most AT‐rich, is a hallmark of cp genomes across land plants. The bias at the third synonymous position (GC3) is particularly pronounced. Nucleotide composition at this position reveals a strong hierarchy: T3 (46.31%–46.74%) > A3 (42.93%–43.47%) > G3 (16.89%–17.42%) > C3 (16.04%–16.40%). This overwhelming preference for T and A over G and C at synonymous sites defines the foundational skew in codon choice.

**TABLE 3:** Codon usage bias parameters of eighteen *Argentina* samples.

Species	GC1/%	GC2/%	GC3/%	GCall/%	T3s/%	C3s/%	A3s/%	G3s/%	ENC	CIA
*Argentina anserina*	46.99	39.28	28.91	38.40	46.59	16.10	43.09	17.35	48.02	0.17
*A. cardotiana*	47.10	39.41	29.04	38.52	46.42	16.27	43.11	17.32	48.23	0.17
*A. fallens*	47.00	39.40	28.79	38.39	46.74	16.04	43.10	17.23	47.99	0.17
*A. festiva*	46.91	39.38	28.65	38.31	46.59	16.09	43.44	16.99	48.08	0.17
*A. gombalana*	47.11	39.37	28.90	38.46	46.59	16.23	43.06	17.24	48.16	0.17
*A. leuconota* var. *leuconota*	46.99	39.42	28.91	38.44	46.59	16.15	43.08	17.30	48.12	0.17
*A. leuconota* var. *brachyphyllaria*	47.03	39.41	28.90	38.45	46.61	16.13	43.09	17.29	48.04	0.17
*A. lineata*	46.89	39.40	28.63	38.30	46.58	16.13	43.47	16.89	48.13	0.17
*A. micropetala*	47.14	39.47	29.12	38.58	46.38	16.34	43.01	17.34	48.42	0.17
*A. microphylla*	47.14	39.47	29.12	38.58	46.38	16.38	43.03	17.33	48.31	0.17
*A. parvula*	47.06	39.42	29.11	38.53	46.40	16.38	43.02	17.32	48.29	0.17
*A. peduncularis*	47.01	39.42	28.93	38.45	46.56	16.13	43.08	17.32	48.13	0.17
*A. phanerophlebia*	47.05	39.43	29.11	38.53	46.36	16.31	43.04	17.38	48.27	0.17
*A. polyphylla*	47.03	39.41	29.09	38.51	46.41	16.34	43.01	17.32	48.24	0.17
*A. smithiana*	47.08	39.37	29.14	38.53	46.46	16.34	42.93	17.42	48.31	0.17
*A. stenophylla* var. *stenophylla*	47.14	39.39	29.17	38.57	46.31	16.40	43.02	17.34	48.41	0.17
*A. stenophylla* var. *emergens*	47.13	39.38	29.05	38.52	46.35	16.39	43.15	17.21	48.39	0.17
*A. taliensis*	47.11	39.36	29.08	38.52	46.34	16.39	43.12	17.25	48.42	0.17

**FIGURE 3:**
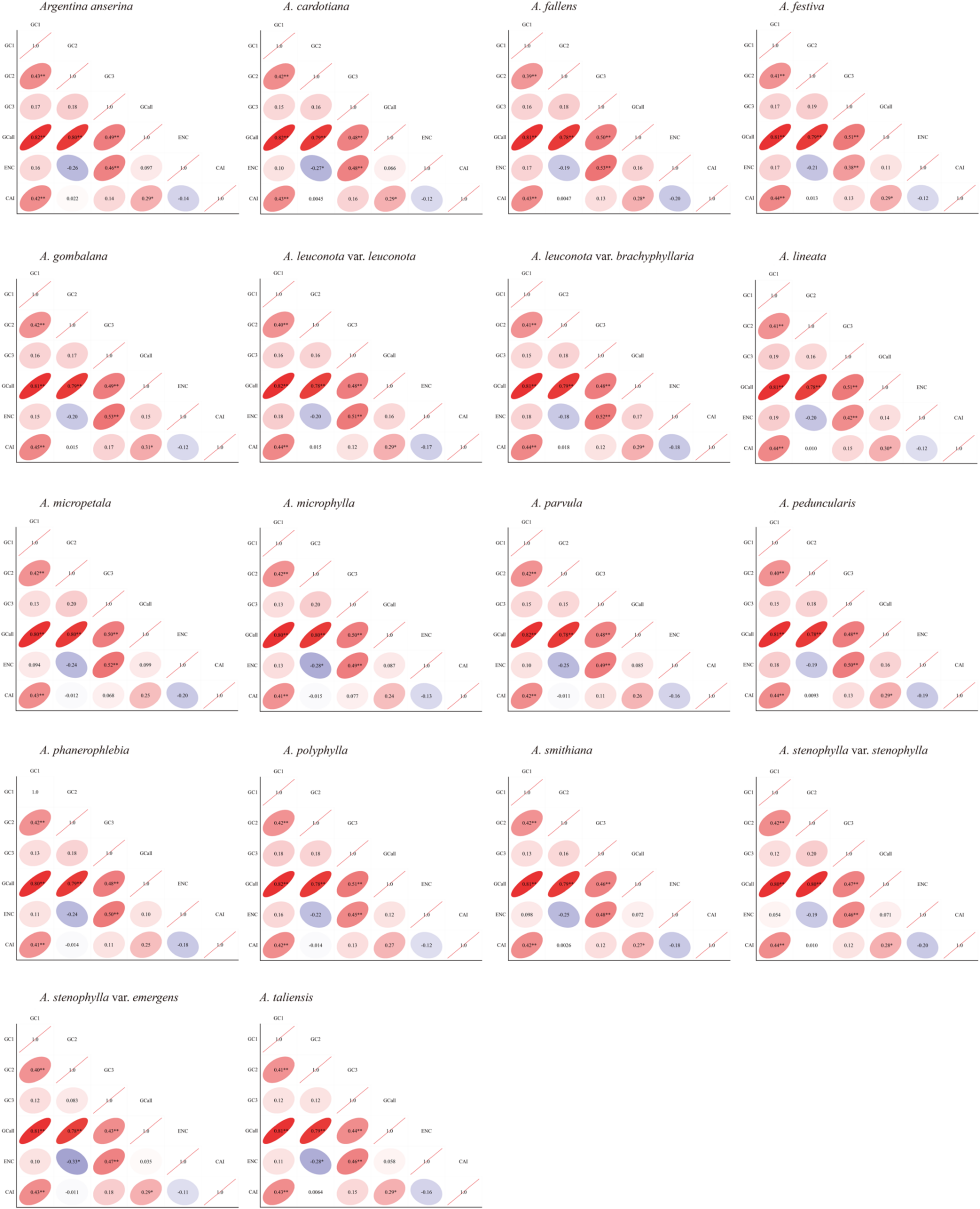
Correlation analysis between parameters of each gene of eighteen *Argentina* samples. Darker color indicates a stronger correlation (positive or negative) between two gene parameters, while lighter color denotes a weaker correlation. (*) means significant correlation at *p* < 0.05; (**) means extremely significant correlation at *p* < 0.01.

Analysis of codon usage patterns among *Argentina* species reveals a consistent AT‐rich signature and weak overall bias. The ENC is consistently high across all species (~48; Table 3), while the CAI—reflecting similarity to highly expressed reference genes—remains uniformly low (~0.17; Table 3), together indicating weak translational selection and moderate expression levels. Correlation analysis revealed a significant correlation between GCall and GC1, GC2, and GC3 (*p* < 0.01; correlation coefficients: 0.46–0.82). Additionally, GC1 and GC2 exhibited a significant correlation (*p* < 0.05), whereas neither GC1 nor GC2 showed a significant correlation with GC3 (Figure 3). This decoupling of GC3 from the functionally constrained first two positions suggests that mutational bias operates most freely at the third codon position. ENC demonstrated a significant positive correlation with GC3 across all species (*p* < 0.01) and a negative correlation with GC2 specifically in *A. cardotiana*. No significant correlation was detected between ENC and either GC1 or GCall (Figure 3). CAI correlates significantly with GC1 across all species and with GCall in most species (except *A. micropetala*, *A. microphylla* , *A. parvula* , *A. polyphylla* , and *A. phanerophlebia*; Figure 3), consistent with its uniformly low values and the overall weak codon bias.

Across the eighteen samples, 29 codons exhibited a RSCU value greater than 1, categorizing them as high‐frequency, “preferred” codons. UUA (Leu) has the highest RSCU, followed by GCU (Ala) (Table S2). Notably, the preference for A‐ or U(T)‐ending codons is predominant: 55.2% of preferred codons end in U, 41.4% in A, and only one codon (UUG for Leu) ends in G. This pattern reinforces the conclusion that the chloroplast genomes of *Argentina*, despite interspecific phylogenetic divergence, share a common codon usage strategy that is heavily biased towards A/T‐rich synonyms.

### 3.4 Analysis of the Forces Shaping CUB

**FIGURE 4:**
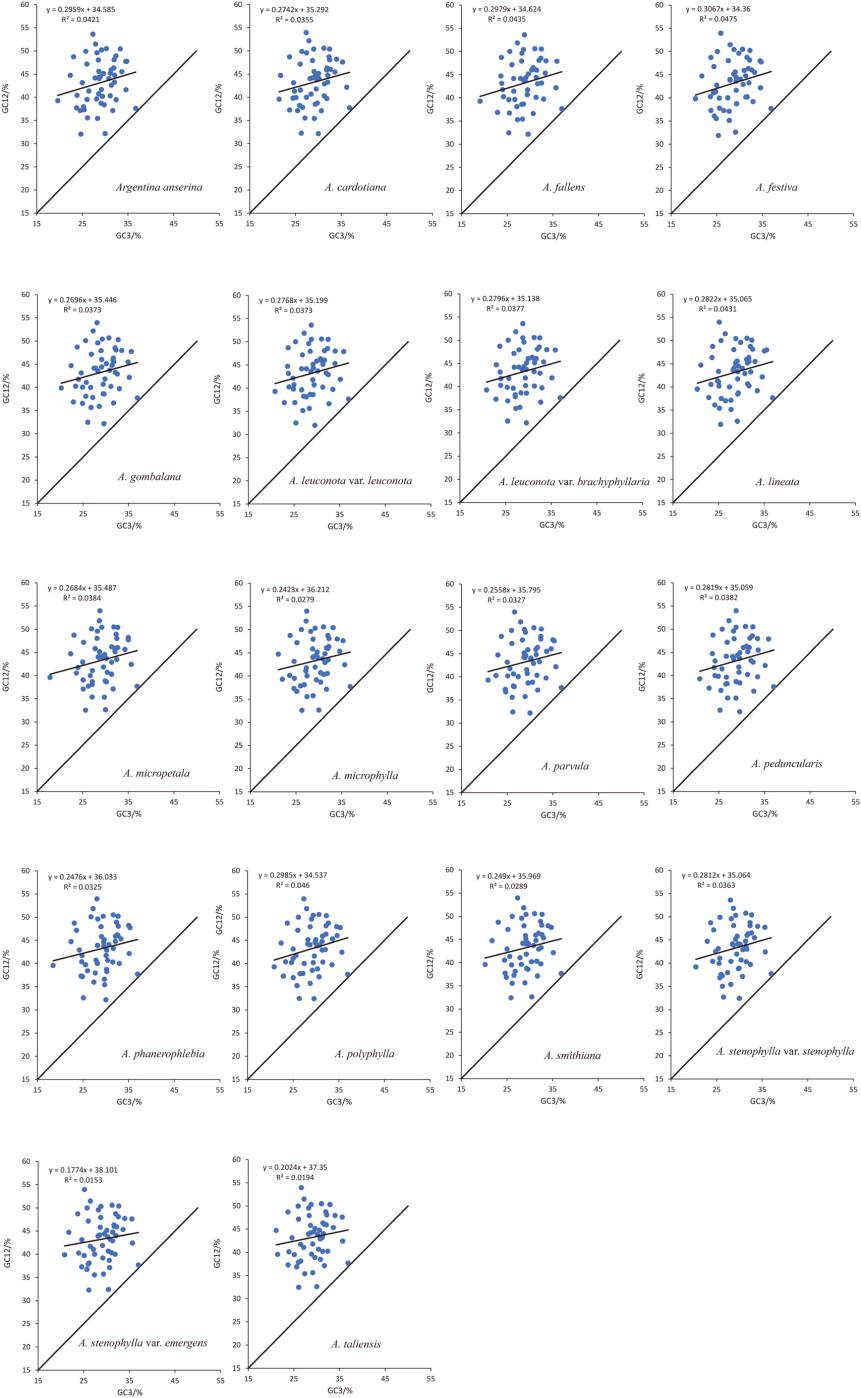
Neutrality plot of eighteen *Argentina* samples.

The neutrality plot, which regresses GC12 against GC3, serves as a critical test (Figure 4). A strong positive correlation is anticipated if mutation pressure is the dominant factor. However, in *Argentina*, the correlation between GC12 and GC3 is weak and non‐significant (*r* = 0.12 to 0.22), with a very low regression slope. This analysis demonstrates that mutation pressure accounts for only 1.53% to 4.75% of the variation in codon usage, while natural selection explains a substantial 95.25% to 98.47%. This finding decisively positions natural selection as the primary determinant of CUB in the cp genomes of all *Argentina* species.

**FIGURE 5:**
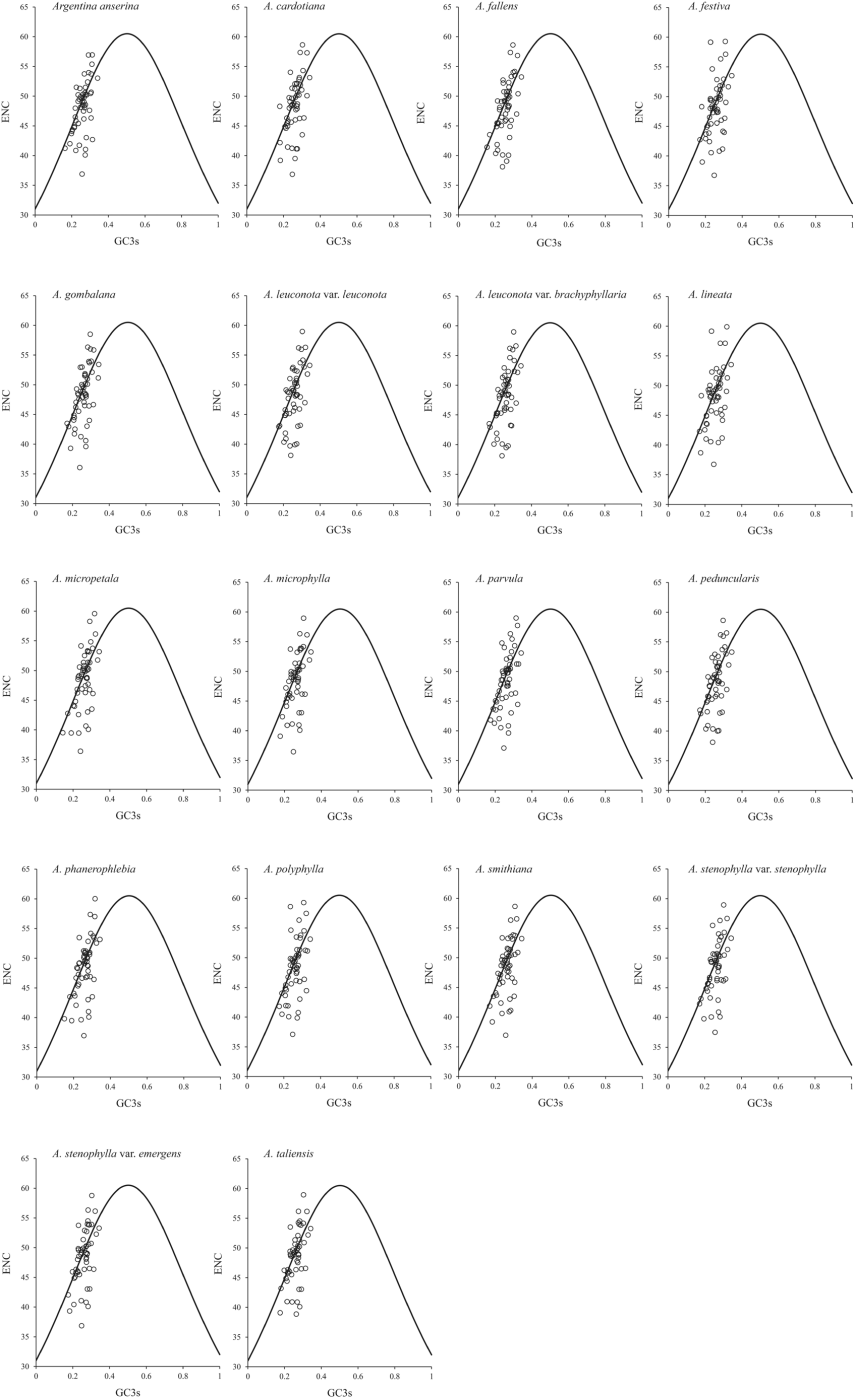
ENC‐plot of eighteen *Argentina* samples.

The ENC‐plot illustrates the observed ENC for each gene against its GC3 content, comparing it to a curve that represents the expected ENC if codon choice were dictated solely by GC3 composition (mutation pressure). In all eighteen *Argentina* samples, the majority of genes fall below the expected curve (Figure 5). This deviation indicates that the observed codon bias is stronger than would be predicted based solely on GC3 content. Such a pattern is a classic signature of natural selection's influence, which constrains codon choice beyond mere mutational tendencies, thereby corroborating the findings of the neutrality plot.

**FIGURE 6:**
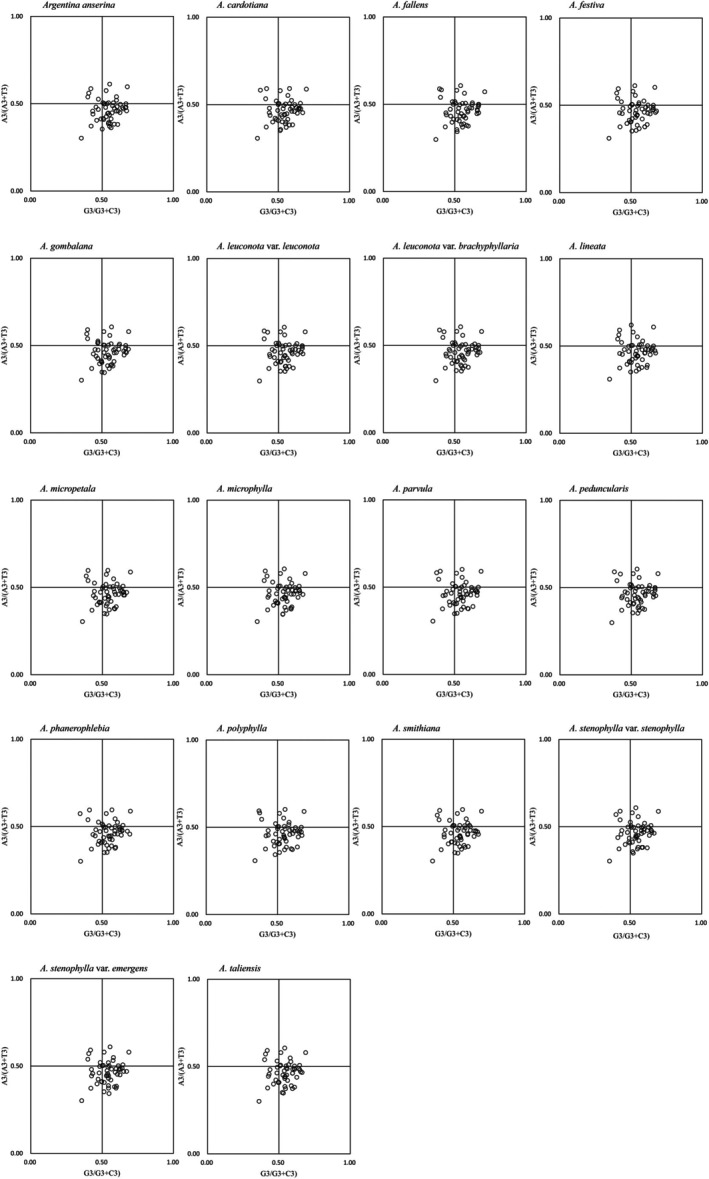
PR2‐plot of eighteen *Argentina* samples.

The PR2 plot analyzes the relationship between A3/(A3 + T3) and G3/(G3 + C3). Under conditions of pure, unbiased mutational pressure, points are expected to cluster at the center (0.5, 0.5), indicating parity between A and T, as well as between G and C at the third codon position. In the eighteen *Argentina* samples, gene points exhibit a marked asymmetric distribution, with a significant concentration in the lower‐right quadrant (where T > A and G > C) (Figure 6). This deviation from parity serves as a robust indicator of the selective forces at play. The preference for T over A and G over C at synonymous sites cannot be accounted for by a symmetric mutational model, thereby strongly implicating natural selection in shaping these preferences. Therefore, the convergent evidence indicated that preferred codons are actively sculpted by natural selection among synonymous options.

### 3.5 Interspecific Divergence in Optimal Codon Repertoires

**FIGURE 7:**
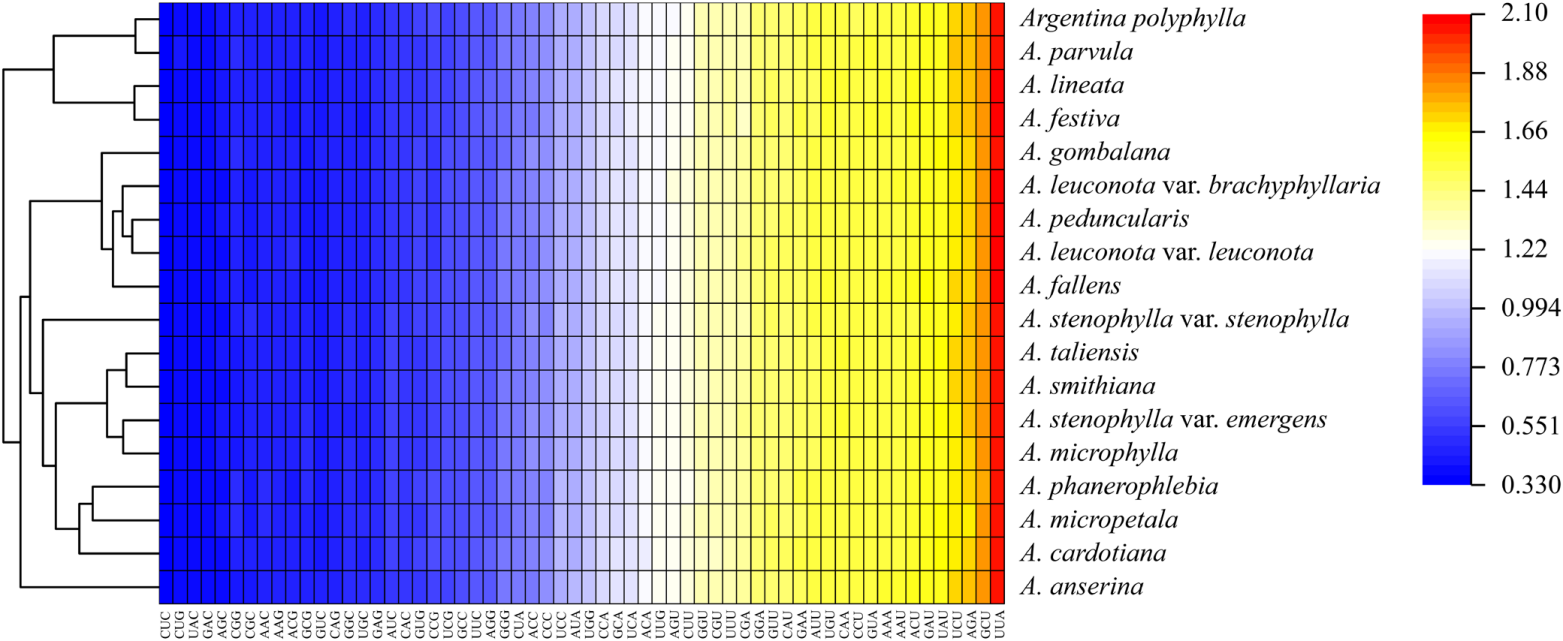
Clustering analysis of RSCU values in eighteen *Argentina* samples.

Our comparative analysis of CUB across the chloroplast genomes of 18 *Argentina* samples reveals a nuanced evolutionary landscape that extends beyond the well‐documented A/T‐rich signature characteristic of plastid genomes. A hierarchical cluster analysis based on RSCU values produced a dendrogram that was broadly congruent with the robust phylogenetic framework derived from whole cp genome sequences (Figure 7). For example, *A. anserina* , which occupies a basal position in Clade I, formed its own distinct cluster, and the four species of Clade V were recovered as a monophyletic group. Conversely, several species displayed RSCU affiliations that departed from their phylogenetic placement. For example, *A. smithiana* , although a member of Clade I, was unexpectedly grouped with species from the more derived Clade IV. Similarly, *A. cardotiana* from Clade III clustered with members of Clade II. This mosaic pattern of phylogenetic concordance and discordance indicates that the evolutionary pressures shaping codon usage are not perfectly aligned with those determining phylogenetic history; rather, lineage‐specific adaptive pressures appear to superimpose upon, and in some cases override, the shared ancestral CUB pattern.

Using stringent criteria (RSCU > 1, ΔRSCU ≥ 0.08), we identified 24 optimal codons corresponding to 17 amino acids (Table 4). Our results revealed a spectrum of conservation and variation: ten amino acids exhibited a single optimal codon. Among these, glutamine (Gln) and glycine (Gly) consistently utilized CAA, and GGU across all 18 species, respectively, indicating strong and shared selective constraints. The optimal codons for other amino acids displayed pronounced interspecific variation. Moreover, seven amino acids were each represented by two optimal codons which fell into two distinct patterns. For Leucine (Leu), valine (Val) and serine (Ser), one codon was universally preferred (e.g., UUA for Leu), whereas the second served as an optimal codon only in a subset of species (e.g., UUG for Leu). The remaining four amino acids with dual optimal codons lacked a universally conserved preference; instead, both codons exhibited variable usage patterns across species.

**TABLE 4:** Comparison of optimal codons of 18 *Argentina* samples.

AA	SP1	SP2	SP3	SP4	SP5	SP6	SP7	SP8	SP9	SP10	SP11	SP12	SP13	SP14	SP15	SP16	SP17	SP18
Gln	CAA**	CAA**	CAA***	CAA*	CAA***	CAA***	CAA***	CAA**	CAA**	CAA***	CAA**	CAA***	CAA**	CAA**	CAA**	CAA**	CAA**	CAA**
Gly	GGU***	GGU***	GGU***	GGU***	GGU***	GGU***	GGU***	GGU***	GGU***	GGU***	GGU***	GGU***	GGU**	GGU***	GGU***	GGU***	GGU***	GGU***
Glu	GAA*	GAA*	—	GAA*	GAA*	—	—	GAA*	GAA*	GAA*	GAA*	—	GAA*	—	GAA*	GAA*	GAA*	—
Cys	—	UGU*	UGU**	UGU*	UGU**	UGU**	—	—	UGU*	—	—	UGU**	UGU***	UGU*	UGU*	UGU*	—	UGU*
Ile	AUU*	AUU*	AUU**	—	AUU*	AUU**	AUU*	AUU*	AUU*	AUU*	—	AUU**	AUU**	—	AUU*	AUU*	AUU*	AUU*
Lys	—	—	AAA*	—	AAA*	AAA*	AAA*	AAA***	AAA*	—	AAA*	AAA*	—	—	AAA*	—	AAA**	AAA*
Phe	—	—	—	—	—	—	—	UUU*	—	—	—	—	UUU*	—	—	—	—	—
Tyr	—	—	—	—	—	—	—	—	UAU*	—	—	—	UAU**	—	—	—	—	—
Asn	—	—	—	—	—	—	—	—	AAU*	—	—	—	—	—	—	—	—	—
Asp	—	—	GAU*	—	—	—	—	—	—	—	—	—	—	—	—	—	—	—
Leu	UUA***	UUA***	UUA**	UUA***	UUA**	UUA**	UUA**	UUA***	UUA***	UUA***	UUA***	UUA**	UUA***	UUA***	UUA***	UUA***	UUA**	UUA***
	—	—	UUG*	UUG*	UUG*	UUG*	UUG*	—	—	—	UUG*	UUG*	—	UUG*	—	—	—	—
Ser	UCU***	UCU***	UCU***	UCU***	UCU***	UCU***	UCU***	UCU***	UCU**	UCU***	UCU***	UCU***	UCU***	UCU***	UCU***	UCU***	UCU***	UCU***
	—	—	—	—	—	—	AGU*	—	AGU*	—	AGU*	—	—	—	—	—	—	AGU*
Val	GUU***	GUU***	GUU**	GUU**	GUU**	GUU**	GUU*	GUU*	GUU*	GUU***	GUU**	GUU**	GUU*	GUU**	GUU**	GUU**	GUU**	GUU*
	GUA*	GUA*	GUA*	GUA*	GUA*	GUA*	GUA*	GUA***	GUA**	—	GUA*	GUA*	GUA**	GUA*	GUA*	GUA*	GUA*	GUA*
Pro	—	—	CCU*	CCU**	CCU*	CCU*	CCU***	CCU***	CCU*	—	CCU**	CCU*	CCU*	CCU**	CCU*	CCU**	CCU*	CCU***
	—	CCA*	—	—	—	—	—	—	—	—	—	—	—	—	—	—	—	—
Thr	—	—	—	ACU***	—	—	ACU***	ACU***	ACU***	—	ACU***	GCU***	ACU***	ACU***	—	—	ACU***	ACU***
	—	ACC*	—	—	—	—	—	—	—	—	—	—	—	—	—	—	—	—
Ala	GCU***	GCU***	GCU***	GCU***	GCU***	GCU***	GCU***	GCU***	GCU**	GCU***	GCU***	—	GCU***	GCU***	GCU***	GCU***	GCU***	GCU***
	—	—	—	—	—	—	—	GCA*	GCA**	—	—	—	—	—	—	—	—	—
Arg	CGU**	CGU*	CGU***	—	CGU***	CGU***	—	—	—	CGU**	—	CGU***	CGU**	—	CGU***	CGU***	CGU**	CGU***
	CGA**	CGA**	CGA*	—	CGA*	CGA*	—	—	CGA***	CGA*	CGA*	CGA*	CGA***	—	CGA**	CGA**	CGA*	CGA*

*Note:* The codons with the star indicate the optimal codons, *ΔRSCU ≥ 0.08; **ΔRSCU ≥ 0.3, ***ΔRSCU ≥ 0.5, SP1‐18: *Argentina anserina, A. cardotiana, A. fallens, A. festiva, A. gombalana, A. leuconota var. leuconota, A. leuconota* var. *brachyphyllaria, A. lineata, A. micropetala, A. microphylla, A. parvula, A. peduncularis, A. phanerophlebia, A. polyphylla, A. smithiana, A. stenophylla* var. *stenophylla, A. stenophylla* var. *emergens, A. taliensis*.

## 4 Discussion

In this study, our whole‐plastome phylogeny strongly supports the monophyly of *Argentina* and resolves five well‐supported clades, corroborating the transfer of *Sibbaldia micropetala* and *S. phanerophlebia* into the genus (Feng et al. 2015). Divergence time estimates place the crown group in the early Miocene (~18.64 Ma) (Li, Zhang, et al. 2024), coinciding with the initial rapid uplift of the Qinghai–Tibetan Plateau and subsequent climatic shifts across the Sino‐Himalayan region (Zhao et al. 2022). This temporal correlation suggests that *Argentina* diversified in tandem with orogenic processes that created new alpine niches, subsequently dispersing into the Malesian archipelago. Notably, the genus exhibits extraordinary morphological plasticity—particularly in stipule architecture and stamen number—that has historically confounded taxonomic delimitation (Feng et al. 2017). Yet this phenotypic lability stands in stark contrast to the extraordinary structural canalization of its plastome: all 18 species retain identical quadripartite architecture and near‐identical gene complements. This dissociation between morphological evolvability and organellar genomic stasis indicates that molecular fine‐tuning, rather than macro‐scale genome restructuring, underpins adaptation in this lineage.

Within this rigid architecture, synonymous codons exhibit surprising dynamism. Neutrality, ENC, and PR2 analyses converge to indicate that natural selection—not mutational pressure—is the principal force shaping CUB, accounting for over 95% of observed variation. This challenges the neutralist paradigm that plastid substitution patterns reflect merely mutational equilibrium and genetic drift (Suzuki and Morton 2016; Liu et al. 2026), revealing a cryptic layer of adaptive evolution beneath structural conservation. Comparative analysis further reveals a dual‐layer pattern in optimal codon repertoires: only Gln and Gly retain universally conserved optimal codons, whereas seven amino acids exhibit dual optimal codons with pronounced interspecific variation. Deeply conserved optimal codons likely reflect ancestral constraints of the plastid translational machinery, while labile assignments suggest ongoing modulation to fine‐tune translational kinetics (Hanson and Coller 2018). In plastids, optimal codons typically match the most abundant tRNA isoacceptors to accelerate ribosome elongation (Kwon et al. 2016); the dual optimal codons in *Argentina* may represent alternative strategies that trade off elongation speed against translational accuracy under abiotic stress (Allan Drummond and Wilke 2009). Such post‐transcriptional adaptation operates independently of protein sequence change, offering a mechanism to sustain energy transduction without altering genomic structure.

Although RSCU‐based hierarchical clustering largely recapitulates the plastome phylogeny, discrete departures in specific lineages suggest that ecological selection has independently modulated codon optima, producing a mosaic pattern not strictly coupled to neutral divergence. However, this analysis was based solely on 53 conserved protein‐coding genes using a distance‐based clustering algorithm, which cannot fully capture the phylogenetic signal of the entire plastome; any inference of lineage‐specific adaptive pressures thus remains preliminary. The sporadic RSCU convergence among distantly related species may reflect independent adaptation to high‐altitude stresses (UV radiation, temperature fluctuations, short growing seasons), favoring convergent codon optima for photosynthetic genes—consistent with evidence from microbial taxa and Zingiberaceae (Lu et al. 2025; Arella et al. 2021). Nevertheless, alternative explanations including mutational biases and stochastic variation cannot be excluded, and whether this variation represents functional adaptation or neutral divergence requires further validation.

The selective dominance over mutation at synonymous sites acquires adaptive meaning in the context of alpine radiation. We hypothesize that high‐altitude physiological pressures—particularly the demand for rapid turnover of damaged photosystem proteins—generate directional selection on synonymous sites, favoring codons that optimize translation elongation rates for stress‐responsive chloroplast genes. This codon‐level versatility may have lowered the energetic cost of colonizing heterogeneous high‐elevation habitats, thereby facilitating the genus‐wide radiation across the Sino‐Himalayan region and the Malay Archipelago. Comparatively, this pattern distinguishes *Argentina* from other Rosaceae: in *Potentilla s.s*. and *Dasiphora*, plastid genomes are similarly structurally conserved, yet codon usage analyses reveal only weak translational selection and homogeneous optimal codon repertoires (Li et al. 2025; Dobe and Paule 2010), suggesting that the alpine transition in *Argentina* intensified selective pressures on synonymous sites. Among alpine groups, *Saussurea* and *Rhodiola* have undergone rapid QTP‐triggered radiation, but their adaptive signatures are documented primarily through protein sequence evolution or gene family expansion (Zhang et al. 2014, 2019; Zhao et al. 2020; Zhang, Kuang, et al. 2023); *Argentina* complements this picture by demonstrating that concerted adaptation can also occur at synonymous positions. Future work integrating nuclear genome skimming, transcriptomic validation, and common‐garden experiments across elevational gradients is essential to establish causal links between codon usage and adaptive evolution in the Himalayan radiation of *Argentina*.

## 5 Conclusions

This study presents the first comprehensive comparative analysis of chloroplast genomes across 18 samples of *Argentina*, integrating phylogenomics with codon usage bias. We establish a robust and fully resolved phylogeny that confirms the monophyly of the genus. Central to our findings is an evolutionary paradox: although the plastome is structurally conserved across *Argentina*, synonymous codons exhibit persistent, lineage‐specific divergence indicative of selective pressures. Natural selection emerges as a dominant force shaping synonymous codon choice, and while RSCU‐based clustering broadly recapitulates the plastome phylogeny, sporadic anomalies suggest that ecological pressures may have independently modulated codon optima in particular lineages. These patterns imply that codon optimization may act as a lineage‐specific adaptive trait contributing to translational efficiency across heterogeneous habitats, rather than merely reflecting shared ancestry. This codon‐level flexibility, operating within a structurally static genomic architecture, likely facilitated the successful radiation of *Argentina* across the Sino‐Himalayan region and the Malay Archipelago, revealing an unanticipated dimension of organellar adaptation. Our findings underscore the utility of chloroplast genome data in resolving complex plant lineages and highlight codon usage bias as an informative, functionally relevant evolutionary trait. Future studies incorporating nuclear genome data and experimental validation will be essential to elucidate the functional consequences of codon optimization and its role in plant adaptation.

## Author Contributions

**Guang‐Nan Zhang:** formal analysis (equal), methodology (equal), writing – original draft (equal). **Li‐Zhen Ling:** formal analysis (equal), methodology (equal), writing – original draft (equal). **Jie Tian:** formal analysis (equal), methodology (equal). **Shu‐Dong Zhang:** conceptualization (lead), writing – review and editing (lead).

## Funding

This research was supported by the Open Subject from Key Laboratory of Qinghai‐Tibetan Plateau Biotechnology of Ministry of Education (2023‐SYS‐04) and Team Building Project of Liupanshui Normal University (LPSSY2023XKTD08).

## Consent

The authors have nothing to report.

## Conflicts of Interest

The authors declare no conflicts of interest.

## Supporting information


**Table S1:** The information of species used for phylogenetic analysis in this study.

**Table S2:** RSCU values of each codon among eighteen *Argentina* species.

## Data Availability

The datasets analyzed during the current study are available from the NCBI database under accession number PX057651 ( *A. stenophylla* var. *stenophylla*), PX057650 (*A. leuconota* var. *leuconota*), and PX057649 ( *A. anserina* ).
